# Regular Consumption of a Flavanol-rich Chocolate can Improve Oxidant Stress in Young Soccer Players

**DOI:** 10.1080/10446670410001722159

**Published:** 2005-03

**Authors:** Cesar G. Fraga, Lucas Actis-Goretta, Javier I. Ottaviani, Fernando Carrasquedo, Silvina B. Lotito, Sheryl Lazarus, Harold H. Schmitz, Carl L. Keen

**Affiliations:** 1 Fisicoquímica-PRALIB Facultad de Farmacia y Bioquímica Universidad de Buenos Aires Argentina; 2 Department of Nutrition University of California Davis CA USA; 3 Analytical and Applied Sciences Mars Inc. Hackettstown NJ USA

## Abstract

The consumption of a diet rich in certain flavonoids, including the flavanol sub-class, has been associated with a reduced risk for vascular disease. We evaluated the effects of the regular consumption (14 d) of a flavanol-containing milk chocolate (FCMC) or cocoa butter chocolate (CBC) on variables related to vascular disease risk, oxidative stress and physical activity. Twenty-eight free-living, young (18–20 years old) male soccer players consumed daily 105 g of FCMC (168 mg of flavanols) or CBC (<5 mg of flavanols), as part of their normal diet. The consumption of FCMC was significantly associated with a decrease in diastolic blood pressure (-5 mm Hg), mean blood pressure (-5 mm Hg), plasma cholesterol (-11%), LDL-cholesterol (-15%), malondialdehyde (-12%), urate (-11%) and lactate dehydrogenase (LDH) activity (-11%), and an increase in vitamin E/cholesterol (+12%). No relevant changes in these variables were associated with CBC consumption. No changes in the plasma levels of (-)-epicatechin were observed following analysis of fasting blood samples. In conclusion, FCMC consumption was associated with changes in several variables often associated with cardiovascular health and oxidant stress. The presence of significant quantities of flavanols in FCMC is likely to have been one of the contributing factors to these results.


*Clinical & Developmental Immunology*, March 2005; 12(1): 11–17

Taylor & Francis

Taylor & Francis Group

### Regular consumption of a flavanol-rich chocolate can improve oxidant stress in young soccer players

CESAR G. FRAGA[1,2], LUCAS ACTIS-GORETTA[1], JAVIER I. OTTAVIANI[1], FERNANDO CARRASQUEDO[1], SILVINA B. LOTITO[1], SHERYL LAZARUS[3], HAROLD H. SCHMITZ[3], & CARL L. KEEN[2]

[1]*Fisicoquímica-PRALIB, Facultad de Farmacia y Bioquímica, Universidad de Buenos Aires, Argentina,* [2]*Department of Nutrition, University of California, Davis, CA, USA, and* [3]*Analytical and Applied Sciences, Mars Inc., Hackettstown, NJ, USA*

**Abstract**
The consumption of a diet rich in certain flavonoids, including the flavanol sub-class, has been associated with a reduced risk for vascular disease. We evaluated the effects of the regular consumption (14 d) of a flavanol-containing milk chocolate (FCMC) or cocoa butter chocolate (CBC) on variables related to vascular disease risk, oxidative stress and physical activity. Twenty-eight free-living, young (18–20 years old) male soccer players consumed daily 105 g of FCMC (168 mg of flavanols) or CBC (< 5 mg of flavanols), as part of their normal diet. The consumption of FCMC was significantly associated with a decrease in diastolic blood pressure (− 5 mm Hg), mean blood pressure (− 5 mm Hg), plasma cholesterol (− 11%), LDL-cholesterol (− 15%), malondialdehyde (− 12%), urate (− 11%) and lactate dehydrogenase (LDH) activity (− 11%), and an increase in vitamin E/cholesterol (+ 12%). No relevant changes in these variables were associated with CBC consumption. No changes in the plasma levels of (−)-epicatechin were observed following analysis of fasting blood samples. In conclusion, FCMC consumption was associated with changes in several variables often associated with cardiovascular health and oxidant stress. The presence of significant quantities of flavanols in FCMC is likely to have been one of the contributing factors to these results.

**Keywords:** *Flavonoids, flavanols, antioxidants, blood pressure, cardiovascular disease*

### Introduction

Increasing effort is being given to the identification of dietary factors that can delay the onset or progression of numerous age-related diseases (Sunde 2001). While essential nutrients are being widely studied in this regard, there is also interest in the evaluation of “non-essential” nutrients that have positive health effects. To a significant extent, this interest has been fueled by epidemiological studies, which suggest that diets rich in plant foods are associated with a reduced risk for vascular disease (Ness and Powles 1997, Bazzano et al. 2002), and possibly, some cancers (Steinmetz and Potter 1996).

Flavonoids are plant-derived compounds that may have multiple beneficial health effects (Hollman 2001, Nijveldt et al. 2001, Knekt et al. 2002). The consumption of a diet rich in flavonoids has been associated with a reduced risk for vascular disease (Kris-Etherton and Keen, 2002). While the inverse association between the risk for vascular disease and dietary flavonoids intake does not prove causality, an increasing amount of experimental data pertaining to certain flavonoids lends support to this association (Middleton et al. 2000).

Cocoa is an example of food that can be a rich dietary source of flavonoids (Lazarus et al. 1999, Arts et al. 1999). High concentrations of flavonoids

Correspondence: C. G. Fraga, Address: Department of Nutrition, University of California, One Shields Avenue, Davis, CA 95616-8669, USA. Tel: 1 530 752 9262. E-mail: cfraga@ffyb.uba.ar

ISSN 1740-2522 print/ISSN 1740-2530 online © 2005 Taylor & Francis Group Ltd.
DOI: 10.1080/10446670410001722159

are present in certain cocoas, predominately as the flavanol monomers (−)-epicatechin (epicatechin) and (+)-catechin (catechin) along with the procyanidins (oligomers of the monomeric flavanol base units) (Adamson et al. 1999). Other potentially rich dietary sources of flavanols include tea, wine, grape juice, apples, peanuts and certain nuts, although growing conditions, post-harvest handling and manufacture can have a significant influence on the flavanol concentration of finished food products (Haslam 1989).

The antioxidant properties of certain flavonoids have been the focus of considerable attention with respect to the hypothesis that flavonoids can reduce the risk for certain oxidant stress-related diseases. The antioxidant actions of the cocoa flavanols (epicatechin and catechin) and procyanidins have been studied *in vitro*. For example, they have been shown to inhibit the rate of LDL-, plasma- and liposome-oxidation (Lotito and Fraga 1998, Lotito and Fraga 2000, Lotito et al. 2000, Pearson et al. 2001). Observations recorded following acute consumption of cocoa or chocolate rich in flavanols have been consistent with *in vitro* studies suggesting that relatively high intake of dietary flavanols can result in an augmentation of the body's oxidative defense system over a short time period (Rein et al. 2000, Wang et al. 2000, Osakabe et al. 2001, Wan et al. 2001). Perhaps, related to any potential modulation of oxidant defense are the observations suggesting that certain flavanol-rich foods may improve biological markers more specifically related to cardiovascular health, including some that are potentially related to blood pressure homeostasis (Diebolt et al. 2001). While the above reports are encouraging, more information is needed regarding the effects of flavanols on oxidative defense mechanisms and other determinants of vascular health.

In this study we evaluated the effects of the regular consumption (14 d) of a flavanol-containing milk chocolate (FCMC) in comparison with a cocoa butter (white) chocolate (CBC) prepared confectionery containing negligible amount of flavanols on several parameters relating to cardiovascular health and oxidant defense. The obtained results showed that several health parameters, including blood pressure, LDL- and total-cholesterol, and glucose levels decreased after the intake of FCMC. These changes were not observed when the same individuals consumed isocaloric amounts of CBC. The consumption of FCMC was also associated with a reduction in plasma level of a lipid oxidation product. Considering the relevance of the blood pressure reducing effect observed after the FCMC consumption, and the ACE inhibition exerted by flavanols (Actis-Goretta et al. 2003), the results of this study induce to further research on the vascular health benefits of flavanols and related oligomers.

### Subjects and methods

### *Subjects and dietary study design*

Twenty-eight healthy, free-living, non-smoking young male adults with no history of heart disease or hemostatic disorders participated in this study was carried out between October and November, 2000 (Table I). All the subjects were active soccer players who were trained at least twice a week, and reported that they had trained at such periodicity at least for the past two years. All of the subjects played in at least one 90-min-match per week in the Argentine Football Association league before and during the study period. The participants gave written informed consent prior to their participation in the study.

During the initial 14-d period, 14 out of the 28 subjects were selected at random to consume FCMC in the form of 105 g of "M&M's"® chocolate confectionery (Mars Incorporated, Recife, Brazil). The other 14 subjects were given 105 g of CBC. At the end of this 14-d period, the two groups were crossed over and subjects consumed the other confectionery for another 14 d. The subjects were free to consume the confectionery at any time during the day. FCMC and CBC were provided in 105 g-coded bags (1-d dose) for 7-d periods. Participants were instructed to abstain from vitamin supplements and flavonoid-rich beverages, including alcohol, for at least 12 h prior to blood collection.

The nutrient composition of the two confectionery products is shown in Table I. By analysis, 105 g of FCMC provided 168 mg of flavanols, of which approximately 39 mg (134 μmol) were the epicatechin and catechin, and, approximately, 126 mg were higher molecular weight procyanidins. CBC contained levels of flavanols under 5 mg per 105 g.

Subjects were fasted at least 8 h prior to blood collection. Venous blood (20 ml) was obtained between 08:00 and 09:00 h, and collected in: (a) three 10-ml plastic tubes containing $K_3EDTA$, potassium oxalate-sodium fluoride, or sodium heparin as anticoagulant, depending on the determination to be performed and (b) one 5-ml glass tube to obtain serum. Blood in the plastic tubes was subjected to low-speed centrifugation (1500*g* at 4°C for 10 min), and plasma was collected and stored at −20°C until analyzed. Blood in the glass tubes was allowed to coagulate at room temperature for 60 min, and then

Table I. Baseline variables determined in the 28 subjects.

| Variable | Value |
|---|---|
| Weight (kg) | 74 ± 1 |
| BMI (kg m$^{-2}$) | 24.1 ± 0.2 |
| Age (y) | 18 ± 1 |
| Hematocrit | 44.0 ± 0.1 |

serum was collected using a plastic pipette. Cardiac frequency and blood pressure were determined immediately before each blood draw using an automatic inflation blood pressure monitor (Omron HEM-712C, Omron Healthcare, Vernon Hills, IL). Immediately after the blood draw, the subjects performed a multistage 20 min shuttle run test to evaluate maximal $VO_2$, as an index of physical performance.

### *Clinical determinations*

Plasma triglycerides, total-, LDL- and HDL-cholesterol, urate, and creatinine kinase (CK) determinations were carried out using commercial kits from Wiener Lab., Rosario, Argentina. Lactate was determined spectrophotometrically using an enzymatic kit (Randox Laboratories, Ltd., Crumlin, UK). Glucose, blood proteins, and the activity of lactate dehydrogenase (LDH), glutamyl pyruvate transferase (GPT), and glutamyl ornithyl transferase (GOT) were determined in a clinical laboratory using current bioassays.

### *Malondialdehyde determination*

Malondiadehyde (MDA) was assayed using frozen heparinized plasma as described (Templar et al. 1999). Essentially, the separation of MDA was carried out in an automated Perkin Elmer HPLC (pump ISS-250, autosampler ISS 200) using a Waters 486 UV–Vis detector set at 532 nm. Chromatography was driven by PE Nelson chromatography software that allowed for data collection and analysis. The analytical column was a reverse phase Supelco Hypersil ODS column (100 × 4.6 × 3 mm). The elution buffer was 65:35 ($VV^{-1}$) 50 mM $KH_2PO_4$—KOH (pH 7.0):methanol.

### *Oxo⁸dG determination*

The extent of blood DNA oxidation was evaluated by quantifying the amount of 8-oxo-2′-deoxyguanosine ($oxo^8dG$), an oxidized DNA base that occurs *in vivo*. DNA from blood cells were isolated following Nakae et al. (1995). After DNA hydrolysis, the measurement of $oxo^8dG$ and dG were carried out by isocratic HPLC with coulometric (guard cell + 400 mV, $E_1$ + 100 mV, $E_2$ + 350 mV) (Coulochem II detector, ESA Inc., Bedford, MA) and UV (254 nm) detections (Helbock et al. 1998). A Supelcosil LC-18DB column (150 × 4.6 × 3 mm) was used, and the mobile phase consisted of 6:94 ($VV^{-1}$) 50 mM sodium acetate (pH 5.5):methanol. Solutions of $oxo^8dG$ and 2′-deoxyguanosine prepared in 1 mM deferoxamine, 20 mM sodium acetate buffer, pH 5.0, were used as standards.

### *Plasma antioxidant activity determination*

Plasma antioxidant activity was determined as total relative antioxidant potency (TRAP) as described previously (Lissi et al. 1995) with minor modifications. Plasma samples (5–10 μl) were assayed for their ability to inhibit the chemiluminescence produced by a mixture of 3 ml of 5.4 mg $ml^{-1}$ of 2,2′-azobis(amidinopropane) in 50 mM sodium phosphate, pH 7.4, and 10 μl of 1 mg $ml^{-1}$ luminol in NaOH 0.1 Eq $l^{-1}$. The chemiluminescence was measured in a liquid scintillation counter (Wallac 1410, Wallac Oy, Turku, Finland). The TRAP value was calculated as the lag time before an increase in the chemiluminescence was observed. This lag time is proportional to the cumulative amount of antioxidants present in the sample (Lissi et al. 1995). A reference lag time was obtained by using a known amount of Trolox (6-hydroxy-2, 5, 7, 8-tetramethoxychroman-2-carboxylic acid) (Aldrich Chem. Co, Milwaukee, WI).

### *Lipid soluble antioxidant determination*

Vitamin E (α-tocopherol), lycopene, β-carotene and ubiquinol-10 (coenzyme Q-10) were determined by HPLC with amperometric detection (Motchnik et al. 1994). The analysis was performed starting from 50 μl of frozen heparinized plasma. The amperometric detector used was a BAS LC-4C 8 (Bioanalytical Systems Inc., West Lafayette, IN) set at + 0.6 V and the analytical column a reverse phase Supelcosil LC-8DB column (33 × 4.6 × 3 mm). The mobile phase consisted of 98:2 ($VV^{-1}$) 20 mM $LiClO_4$:methanol.

### *Epicatechin and catechin determination*

A 200 μl aliquot of heparinized plasma was mixed with 20 μl of a solution containing 0.2 g $ml^{-1}$ ascorbic acid, 1 mg $ml^{-1}$ EDTA, and 20 μl of beta-glucuronidase suspension (about 2000 units of glucuronidase activity and 80 units of sulfatase activity), and vortexed for 30 s. Following a 45 min-incubation at 37°C, the samples were treated as reported (Rein et al. 2000), and epicatechin and catechin determined by HPLC with coulometric detection using a reverse phase Alltech Alltima C-18 column (150 × 4.6 × 3 mm). The mobile phase consisted of 30:70 ($VV^{-1}$) 50 mM sodium acetate (pH 5.5):methanol. The coulometric detector was an ESA Coulochem II (ESA Inc., Bedford, MA) equipped with an analytical cell, ESA 5111, set at + 0.8 mV.

### *Statistical analysis*

Results in the text and tables are expressed as mean ± SEM. Changes between the baseline (day 0) and day 14 were examined by paired *t* test. Regression analysis was used to compare associations between the

Table II. Total nutrient composition of FCMC and CBC.

| Nutrient | FCMC (%) | CBC (%) |
|---|---|---|
| Fat | 21.3 | 21.6 |
| Lactose | 4.4 | 8.0 |
| Sucrose | 54.5 | 60.2 |
| Fiber | 2.5 | nd |
| Ash | 1.6 | 1.5 |
| Caffeine | 0.017 | nd |
| Theobromine | 0.17 | nd |
| Moisture | 1.7 | 1.3 |
| Protein | 4.3 | 4.1 |
| Minerals | 0.6 | 0.5 |
| Cholesterol | 0.014 | 0.014 |
| Flavanols | 0.16 | nd |

Data provided by Mars, Incorporated.

variables. Statistical significance was assessed at the 5% level. Analysis was performed using routines available in Statview for Windows v 5.0.1. (SAS Institute Inc., Cary, NC).

### Results

Participant characteristics are shown in Table II, and there were no significant differences in the analyzed variables between the two groups. Twenty-seven of the 28 enrolled subjects completed the study. All of the subjects reported that they consumed the appropriate amount of product throughout the study period. None of the subjects reported adverse effects and/or changes in their athletic performance related to the confectionery consumption. All of the subjects maintained their weight during the 28-d test period study. Neither epicatechin nor catechin was detected in the plasma samples through the study.

The results observed in several variables associated with cardiovascular health are shown in Table III. Relative to baseline values, diastolic and mean blood pressure, blood levels of total cholesterol, LDL-cholesterol and glucose were lower in the subjects after they consumed FCMC for 14 d as compared with baseline values. No differences were observed in the same variables determined before and after the consumption of CBC (Table III).

The effects of the dietary treatments on oxidative stress variables are shown in Table IV. Relative to baseline values, plasma levels of malondialdehyde were decreased by 12% after the 14-d of FCMC intake, relative to baseline values ($p < 0.001$). In contrast, after the 14-d period of CBC intake, plasma malondialdehyde levels were increased 10% ($p < 0.05$). Plasma vitamin E (α-tocopherol) levels were not influenced by the diets. However, the vitamin E/cholesterol and vitamin E/LDL ratios were increased by 12% ($p < 0.01$) and 14% ($p < 0.03$), respectively, after FCMC consumption. No changes were observed in vitamin E levels and vitamin E/cholesterol and vitamin E/LDL ratios after CBC consumption. Plasma β-carotene concentrations were 13% lower after the intake of CBC ($p < 0.05$). Plasma urate levels after 14 d on FCMC were 11% lower than baseline values ($p = 0.05$). The other oxidative stress markers assessed in this study (blood levels of oxo$^8$dG and plasma TRAP, lycopene, and coenzyme Q-10 concentrations) were not influenced by the consumption of FCMC or CBC.

The effects of the chocolate consumption on select physiological exercise variables are shown in Table V. Relative to baseline values, after CBC consumption, blood lactate values decreased by 22% ($p < 0.03$). Plasma LDH activity was significantly lower (11%) than baseline activity in the subjects after the intake of FCMC ($p < 0.001$). There was no effect of the dietary treatments on plasma GOT, GPT, or CK activities or in maximal $VO_2$.

### Discussion

We observed that daily intake of FCMC for 14 d favorably influenced several variables associated with cardiovascular health and oxidant defense in well-conditioned young men. The contribution of FCMC to the total caloric intake of the subjects was about 11% considering an average total caloric intake of about 4000 kCal per day. Presumably, the addition of

Table III. Changes in physiological variables observed after 14 days of FCMC or CBC consumption.

| | FCMC | | | CBC | | |
|---|---|---|---|---|---|---|
| Variable | Baseline | Day 14 | $p$ | Baseline | Day 14 | $p$ |
| Cardiac frequency ($min^{-1}$) | 59 ± 2 | 61 ± 2 | 0.40 | 62 ± 2 | 57 ± 2 | 0.05 |
| Systolic blood pressure (mm Hg) | 123 ± 3 | 117 ± 2 | 0.06 | 123 ± 3 | 121 ± 2 | 0.54 |
| Diastolic blood pressure (mm Hg) | 72 ± 2 | 67 ± 2 | 0.01 | 71 ± 2 | 70 ± 2 | 0.74 |
| Mean blood pressure (mm Hg) | 89 ± 2 | 84 ± 2 | 0.008 | 88 ± 2 | 87 ± 2 | 0.64 |
| Cholesterol ($mg\,dl^{-1}$) | 167 ± 6 | 149 ± 4 | 0.009 | 156 ± 5 | 157 ± 5 | 0.69 |
| LDL-cholesterol ($mg\,dl^{-1}$) | 107 ± 5 | 91 ± 4 | 0.02 | 101 ± 5 | 96 ± 4 | 0.47 |
| HDL-cholesterol ($mg\,dl^{-1}$) | 35 ± 2 | 33 ± 1 | 0.20 | 33 ± 1 | 35 ± 2 | 0.36 |
| LDL/HDL | 3.2 ± 0.2 | 2.8 ± 0.1 | 0.14 | 3.1 ± 0.2 | 2.9 ± 0.2 | 0.37 |
| Triglycerides ($mg\,dl^{-1}$) | 92 ± 12 | 81 ± 5 | 0.30 | 86 ± 5 | 92 ± 11 | 0.48 |

Values are mean ± SEM. $p$ is obtained from the comparison of day 14 and day 0 values (paired $t$-test). There were no significant differences between baseline values for any of the variables determined.

Table IV. Changes in oxidative stress variables observed after 14 days of FCMC or CBC consumption.

| Variable | FCMC Day 0 | FCMC Day 14 | FCMC *p* | CBC Day 0 | CBC Day 14 | CBC *p* |
|---|---|---|---|---|---|---|
| Malondialdehyde (nM) | 85 ± 3 | 75 ± 3 | 0.001 | 80 ± 3 | 88 ± 2 | 0.04 |
| Oxo$^8$dG (bases per $10^5$ dG) | 1.8 ± 0.1 | 1.8 ± 0.2 | 0.95 | 1.6 ± 0.3 | 1.8 ± 0.2 | 0.39 |
| TRAP (s) | 325 ± 27 | 282 ± 13 | 0.20 | 304 ± 19 | 322 ± 24 | 0.72 |
| Vitamin E (μM) | 28.4 ± 1.5 | 28.5 ± 1.1 | 0.83 | 27.8 ± 1.3 | 28.8 ± 1.3 | 0.28 |
| Vitamin E/cholesterol | 0.17 ± 0.01 | 0.19 ± 0.01 | 0.01 | 0.18 ± 0.01 | 0.19 ± 0.01 | 0.91 |
| Vitamin E/LDL | 0.28 ± 0.02 | 0.32 ± 0.01 | 0.02 | 0.29 ± 0.02 | 0.31 ± 0.02 | 0.40 |
| β-Carotene (μM) | 0.40 ± 0.09 | 0.38 ± 0.05 | 0.86 | 0.39 ± 0.06 | 0.34 ± 0.05 | 0.04 |
| Lycopene (μM) | 0.46 ± 0.05 | 0.47 ± 0.05 | 0.54 | 0.46 ± 0.05 | 0.46 ± 0.05 | 0.98 |
| Coenzyme Q-10 (μM) | 0.42 ± 0.03 | 0.40 ± 0.03 | 0.74 | 0.41 ± 0.03 | 0.43 ± 0.04 | 0.54 |
| Urate (M) | 0.28 ± 0.01 | 0.25 ± 0.02 | 0.05 | 0.25 ± 0.02 | 0.26 ± 0.02 | 0.54 |

Values are mean ± SEM. *p* is obtained from the comparison of day 14 and day 0 values (paired *t*-test). There were no significant differences between baseline values for any of the variables determined.

calories provided by FCMC was offset by reduction in the consumption of other foods as body weight during the 28-d test period was unaffected.

High blood pressure is a risk factor for numerous diseases (stroke, cardiovascular disease) while reductions in blood pressure, even within normal range may be beneficial. FCMC consumption was associated with a modest, but significant, reduction in blood pressure. This effect was not an acute effect as shown by the results from our laboratory in a group of young adults (21–23 years) (unpublished). The change in blood pressure was not observed when the subjects consumed the CBC. A similar study carried out in aged people, showed a similar effect of the consumption of chocolate on blood pressure (Taubert et al. 2003). Flavonoid-rich foods have demonstrated the potential to influence endothelial function in humans (Duffy et al. 2001, Heiss et al. 2003), and to modulate nitric oxide synthesis and/or bioavailability in and around the vascular bed (Karim et al. 2000, Freedman et al. 2001). Given that the main difference between the two products consumed in this study was the flavanol content of FCMC, it is therefore, possible that the favorable blood pressure changes recorded in this study were ultimately mediated through an action of the flavanols, or their metabolites. But, it is also possible to consider as an alternative mechanism, the inhibition of the angiotensin converting enzyme (ACE) activity. In this regard, we have shown that flavanols extracted from cocoa, including epicatechin and the dimer and hexamer procyanidin fractions, inhibit ACE activity *in vitro* (Actis-Goretta et al. 2003). Furthermore, it was reported that the intake of pomegranate juice, which is rich in flavonoids, lowered blood pressure in a group of hypertensive patients (Aviram and Dornfeld 2001) and that the oral administration of wine polyphenols decreased blood pressure in rats (Diebolt et al. 2001). More research on this potential interaction needs to be undertaken to confirm the plausibility of these vascular actions *in vivo*.

FCMC consumption was also associated with a decrease in blood levels of total-cholesterol and LDL-cholesterol, but not with significant changes in HDL-cholesterol and total plasma triglyceride levels. These observations are consistent with the findings of a study of Japanese women that showed an inverse correlation between flavonoid intake and plasma LDL-cholesterol (Arai et al. 2000). We also noted that the above findings are in contrast with those of Wan et al. (2001), who observed no changes in total and LDL-cholesterol, and a subtle increase in the level of HDL-cholesterol (4%), after the consumption of a mixture of dark chocolate and cocoa powder.

Table V. Changes in physiological variables observed after 14 days of FCMC or CBC consumption.

| Variable | FCMC Baseline | FCMC Day 14 | FCMC *p* | CBC Baseline | CBC Day 14 | CBC *p* |
|---|---|---|---|---|---|---|
| Lactate | 9.7 ± 5 | 91 ± 4 | 0.55 | 12.9 ± 0.9 | 10.0 ± 0.6 | 0.03 |
| Lactate dehydrogenase ($\mathrm{U\,l^{-1}}$) | 397 ± 14 | 354 ± 12 | 0.001 | 373 ± 11 | 362 ± 10 | 0.26 |
| GOT ($\mathrm{U\,l^{-1}}$) | 25.1 ± 3.1 | 23.1 ± 1.4 | 0.48 | 22.6 ± 1.1 | 25.0 ± 3.0 | 0.41 |
| GPT ($\mathrm{U\,l^{-1}}$) | 23.9 ± 2.8 | 23.9 ± 2.5 | 0.93 | 21.5 ± 1.5 | 24.7 ± 3.0 | 0.12 |
| Creatinine kinase ($\mathrm{U\,l^{-1}}$) | 232 ± 22 | 223 ± 19 | 0.56 | 203 ± 20 | 235 ± 20 | 0.12 |
| $VO_2$ ($\mathrm{ml\,kg^{-1}\,min^{-1}}$) | 55.2 ± 0.8 | 55.2 ± 0.7 | 0.73 | 54.7 ± 0.6 | 57.3 ± 0.9 | 0.10 |

Values are mean ± SEM. *p* is obtained from the comparison of day 14 and day 0 values, using paired *t*-test. There were no significant differences between baseline values for any of the variables determined.

In addition, it is important to consider that there is a massive amount of literature indicating that the ingestion of cocoa butter, or chocolate containing cocoa butter, is not associated with changes in any blood cholesterol parameters (Wan et al. 2001, Mathur et al. 2002). This reinforces our observation that CBC consumption had a neutral effect on blood cholesterol, but is not in line with the decrease in total- and LDL-cholesterol after FCMC consumption that we report here. From these results we speculate that the high content of flavanols and procyanidins in the chocolate used in our study could be responsible for the decrease in total- and LDL-cholesterol.

With respect to oxidative stress, plasma malondialdehyde levels were lower after 14 d of FCMC consumption compared to baseline. This result agrees with previous reports of an inverse association between plasma thiobarbituric acid reactive substances (TBARS) concentrations and plasma epicatechin concentrations in subjects given different amounts of flavanol-rich chocolate (Rein et al. 2000, Wang et al. 2000). Similarly, decreased rates of *ex-vivo* LDL oxidation have been reported in individuals after the consumption of flavanol-rich chocolate (Osakabe et al. 2001, Wan et al. 2001). The other studied marker of oxidative stress oxo$^8$dG as well as the oxidant defenses (TRAP, vitamin E, β-carotene, lycopene, and ubiquinol-10) followed in this study were unaffected by the dietary treatments. It is worth considering that the observed higher ratios of vitamin E to cholesterol and to LDL after the consumption of the FCMC are suggestive of improved antioxidant protection of plasma cholesterol and LDL. However, it should be considered that this relative increase could be associated with the decrease in cholesterol concentration. A potential relationship between oxidative stress and blood pressure can not be disregarded. This may be related to a direct scavenging effect by the flavanols and procyanidins, and/or with the regulation of the oxidants and/or nitric oxide steady state in vascular associated cells (Diebolt et al. 2001, Leikert et al. 2002).

It is interesting to note that we did not detect epicatechin or procyanidins in the plasma after the 14 d consumption of FCMC. Similar results were reported by Osakabe et al. (2001), although in that study an increased urinary excretion of epicatechin was observed 1 and 2 weeks after cocoa consumption. The absence of epicatechin and/or procyanidins was expected considering that we drew the blood after a 8 h-fast, and the majority of absorbed epicatechin and dimer are cleared from the blood by that time (Rein et al. 2000, Holt et al. 2002). Absence of epicatechin in the plasma could be responsible for the absence of an increase in the total plasma antioxidant activity (TRAP) in the fasted blood samples when subjects were consuming FCMC. Thus, while epicatechin is an example of a nutrient that is only transiently detected in blood, the potential health benefits of flavanols suggested both *in vitro* and following consumption by humans emphasizes the need for further study to determine if other tissues might take up these bioactive compounds. In this regard, we have observed that flavanols, especially, the dimer fraction can influence cell signaling in Jurkat cells (Mackenzie et al. 2004). Thus, additional research will also be needed to determine how individual cells within these tissues might accumulate and/or metabolize the flavanols.

In conclusion, we observed several changes in markers associated with cardiovascular health and oxidant defense following consumption of FCMC by physically active young men. Particularly striking were the observations that FCMC favorably modulated both blood pressure and blood cholesterol levels as compared to CBC consumption. Given, that we previously reported that select flavanols inhibit ACE *in vitro* (Actis-Goretta et al. 2003), this may therefore represent another mechanism associated with cardiovascular health that flavanols can target following consumption and absorption. Finally, some parameters related to oxidant defense were favorably modulated, and this is consistent with the well-documented strong antioxidant potential of flavanols. Taken together, the results from this study provide further supporting evidence for the hypothesis that the regular consumption of flavanol-rich foods can positively contribute to cardiovascular health.

### Acknowledgements

Supported with grants from the University of Buenos Aires (UBACYT B-042) and the ANPCYT (PICT 08951/2001).

### References

Actis-Goretta L, Ottaviani JI, Keen CL, Fraga CG. 2003. Inhibition of angiotensin converting enzyme (ACE) activity by flavan-3-ols and procyanidins. FEBS Lett 555:597–600.

Adamson GE, Lazarus SA, Mitchell AE, et al. 1999. HPLC method for the quantification of procyanidins in cocoa and chocolate samples and correlation to total antioxidant capacity. J Agric Food Chem 47:4184–4188.

Arai Y, Watanabe S, Kimira M, Shimoi K, Mochizuki R, Kinae N. 2000. Dietary intakes of flavonols, flavones and isoflavones by Japanese women and the inverse correlation between quercetin intake and plasma LDL cholesterol concentration. J Nutr 130:2243–2250.

Arts IC, Hollman PC, Kromhout D. 1999. Chocolate as a source of tea flavonoids. Lancet 354:488.

Aviram M, Dornfeld L. 2001. Pomegranate juice consumption inhibits serum angiotensin converting enzyme activity and reduces systolic blood pressure. Atherosclerosis 158:195–198.

Bazzano LA, He J, Ogden LG, et al. 2002. Fruit and vegetable intake and risk of cardiovascular disease in US adults: The first National Health and Nutrition Examination Survey Epidemiologic Follow-up Study. Am J Clin Nutr 76:93–99.

Diebolt M, Bucher B, Andriantsitohaina R. 2001. Wine polyphenols decrease blood pressure, improve NO vasodilatation, and induce gene expression. Hypertension 38:159–165.

Duffy SJ, Keaney JF, Jr., Holbrook M, et al. 2001. Short- and long-term black tea consumption reverses endothelial dysfunction in patients with coronary artery disease. Circulation 104:151–156.

Freedman JE, Parker C, III, Li L, et al. 2001. Select flavonoids and whole juice from purple grapes inhibit platelet function and enhance nitric oxide release. Circulation 103:2792–2798.

Haslam E. 1989. Practical polyphenolics. From structure to molecular recognition and physiological action. Cambridge, UK: Cambridge University Press.

Heiss C, Dejam A, Kleinbongard P, Schewe T, Sies H, Kelm M. 2003. Vascular effects of cocoa rich in flavan-3-ols. J Am Med Assoc 290:1030–1031.

Helbock HJ, Beckman KB, Shigenaga MK, et al. 1998. DNA oxidation matters: The HPLC-electrochemical detection assay of 8-oxo-deoxyguanosine and 8-oxo-guanine. Proc Natl Acad Sci USA 95:288–293.

Hollman PC. 2001. Evidence of health benefits of plant phenols: Local or systemic effects. J Sci Food Agric 81:842–852.

Holt RR, Lazarus SA, Sullards MC, et al. 2002. Procyanidin dimer B2 [epicatechin-(4beta-8)-epicatechin] in human plasma after the consumption of a flavanol-rich cocoa. Am J Clin Nutr 76:798–804.

Karim M, McCormick K, Kappagoda CT. 2000. Effects of cocoa extracts on endothelium-dependent relaxation. J Nutr 130:2105S–2108S.

Knekt P, Kumpulainen J, Jarvinen R, et al. 2002. Flavonoid intake and risk of chronic diseases. Am J Clin Nutr 76:560–568.

Kris-Etherton PM, Keen CL. 2002. Evidence that the antioxidant flavonoids in tea and cocoa are beneficial for cardiovascular health. Curr Opin Lipidol 13:41–49.

Lazarus SA, Adamson GE, Hammerstone JF, Schmitz HH. 1999. High-performance liquid chromatography/mass spectrometry analysis of proanthocyanidins in foods and beverages. J Agric Food Chem 47:3693–3701.

Leikert JF, Rathel TR, Wohlfart P, Cheynier V, Vollmar AM, Dirsch VM. 2002. Red wine polyphenols enhance endothelial nitric oxide synthase expression and subsequent nitric oxide release from endothelial cells. Circulation 106:1614–1617.

Lissi E, Salim-Hanna M, Pascual C, del Castillo MD. 1995. Evaluation of total antioxidant potential (TRAP) and total antioxidant reactivity from luminol-enhanced chemiluminescence measurements. Free Radic Biol Med 18:153–158.

Lotito SB, Fraga CG. 1998. (+)-Catechin prevents human plasma oxidation. Free Radic Biol Med 24:435–441.

Lotito SB, Fraga CG. 2000. Catechins delay lipid oxidation and alpha-tocopherol and beta-carotene depletion following ascorbate depletion in human plasma. Proc Soc Exp Biol Med 225:32–38.

Lotito SB, Actis-Goretta L, Renart ML, et al. 2000. Influence of oligomer chain length on the antioxidant activity of procyanidins. Biochem Biophys Res Commun. 276:945–951.

Mackenzie GG, Carrasquedo F, Delfino JM, Keen CL, Fraga CG, Oteiza PI. 2004. Epicatechin, catechin, and dimeric procyanidins inhibit PMA-induced NF-kappaB activation at multiple steps in Jurkat T cells. FASEB J 18:167–169 (Epub 2003, Nov 20).

Mathur S, Devaraj S, Grundy SM, Jialal I. 2002. Cocoa products decrease low density lipoprotein oxidative susceptibility but do not affect biomarkers of inflammation in humans. J Nutr 132:3663–3667.

Middleton EJ, Kandaswami C, Theoharides TC. 2000. The effects of plant flavonoids on mammalian cells: Implications for inflammation, heart disease, and cancer. Pharmacol Rev 52:673–751.

Motchnik PA, Frei B, Ames BN. 1994. Measurement of antioxidants in human blood plasma. Methods Enzymol 234:269–279.

Nakae D, Mizumoto Y, Kobayashi E, Noguchi O, Konishi Y. 1995. Improved genomic/nuclear DNA extraction for 8-hydroxydeoxyguanosine analysis of small amounts of rat liver tissue. Cancer Lett 97:233–239.

Ness AR, Powles JW. 1997. Fruit and vegetables, and cardiovascular disease: A review. Int J Epidemiol 26:1–13.

Nijveldt RJ, van Nood E, van Hoorn DE, Boelens PG, van Norren K, van Leeuwen PA. 2001. Flavonoids: A review of probable mechanisms of action and potential applications. Am J Clin Nutr 74:418–425.

Osakabe N, Baba S, Yasuda A, et al. 2001. Daily cocoa intake reduces the susceptibility of low-density lipoprotein to oxidation as demonstrated in healthy human volunteers. Free Radic Res 34:93–99.

Pearson DA, Schmitz HH, Lazarus SA, Keen CL. 2001. Inhibition of *in vitro* low-density lipoprotein oxidation by oligomeric procyanidins present in chocolate and cocoas. Methods Enzymol 335:350–360.

Rein D, Lotito S, Holt RR, Keen CL, Schmitz HH, Fraga CG. 2000. Epicatechin in human plasma: *in vivo* determination and effect of chocolate consumption on plasma oxidation status. J Nutr 130:2109S–2114S.

Steinmetz KA, Potter JD. 1996. Vegetables, fruit, and cancer prevention: A review. J Am Diet Assoc 96:1027–1039.

Sunde RA. 2001. Research needs for human nutrition in the post-genome-sequencing era. J Nutr 131:3319–3323.

Taubert D, Berkels R, Roesen R, Klaus W. 2003. Chocolate and blood pressure in elderly individuals with isolated systolic hypertension. J Am Med Assoc 290:1029–1030.

Templar J, Kon SP, Milligan TP, Newman DJ, Raftery MJ. 1999. Increased plasma malondialdehyde levels in glomerular disease as determined by a fully validated HPLC method. Nephrol Dial Transplant 14:946–951.

Wan Y, Vinson JA, Etherton TD, Proch J, Lazarus SA, Kris-Etherton PM. 2001. Effects of cocoa powder and dark chocolate on LDL oxidative susceptibility and prostaglandin concentrations in humans. Am J Clin Nutr 74:596–602.

Wang JF, Schramm DD, Holt RR, et al. 2000. A dose-response effect from chocolate consumption on plasma epicatechin and oxidative damage. J Nutr 130:2115S–2119S.

